# Safety and efficacy of the ROCK-2-inhibitor Belumosudil in cGvHD treatment - a retrospective, German-Swiss multicenter real-world data analysis

**DOI:** 10.1038/s41409-024-02507-9

**Published:** 2025-01-14

**Authors:** Silke Heidenreich, Katharina Egger-Heidrich, Jörg P. Halter, Lasse Jost, Friedrich Stölzel, Markus Perl, Alexander Denk, Matthias Edinger, Wolfgang Herr, Nicolaus Kröger, Daniel Wolff, Francis Ayuk, Matthias A. Fante

**Affiliations:** 1 Department of Stem Cell Transplantation, University Hospital Hamburg-Eppendorf, Hamburg, Germany; 2 Department of Internal Medicine I, University Hospital Carl Gustav Carus, Dresden, Germany; 3 Division of Hematology, University Hospital Basel, Basel, Switzerland; 4 Division of Stem Cell Transplantation and Cellular Immunotherapies, University Hospital Schleswig-Holstein, Kiel, Germany; 5 Department of Internal Medicine III, University Hospital Regensburg, Regensburg, Germany

**Keywords:** Haematological diseases, Stem-cell therapies

## Abstract

Belumosudil is a first in class ROCK2-inhibitor approved by the FDA for the 3rd line treatment of chronic graft-versus-host disease (cGvHD). In this retrospective real-world analysis, we report safety and efficacy data of belumosudil treatment from 5 German/Swiss transplant centers. A total of 33 adult patients (median age 59 years) with moderate (*n* = 2) or severe (*n* = 31) cGvHD were treated on individual request due to lack of EMA approval. The patient cohort had a long history of cGvHD (median 44 months) and was heavily pretreated (median 4 prior lines). The overall response rate was 42% (95%CI, 25–60%) including organ responses in all organs except the liver (*n* = 2). The median time to response was 3 months (range, 1–9 months) and 8 of 14 patients (57%) had a durable response at last follow-up. One-third of patients had at least a 50% reduction in concomitant corticosteroid dosage. Median failure-free survival and median overall survival were 16.5 and 23.1 months, respectively. Adverse events ≥CTCAE grade 3 were reported in 27% of patients, with a predominance of infectious events, including one fatal course. The results are consistent with previous prospective trials including a favorable safety profile, while acknowledging the challenges of a heavily pretreated patient cohort.

## Introduction

Chronic graft-versus-host disease (cGvHD) is a major complication after allogeneic hematopoietic stem cell transplantation (alloHSCT), affecting approximately half of the patients and representing a major cause of late non-relapse mortality (NRM) [1–4]. Corticosteroids (CS) are the established first-line treatment for cGvHD, but 50% of patients are steroid refractory. Ruxolitinib has been approved by the European Medicines Agency (EMA) for second-line treatment and achieves overall response rates (ORR) of 50% [5, 6]. Treatment options besides ruxolitinib in the second-line setting include the Food and Drug Association (FDA-), but not EMA- approved ibrutinib, which has limitations due to high rates of adverse events but yields ORR of up to 74% [7–9]. Recently, the colony-stimulating factor 1 receptor (CSF1-R) inhibitor axatilimab was approved by the FDA for third-line treatment but has not yet been applied outside of clinical trials [10, 11]. Despite recent approvals, the treatment of advanced cGvHD remains challenging with only one third of patients being off immunosuppression, alive, and free from malignancies at five years after onset of cGvHD [3].

In July 2021, belumosudil, a selective inhibitor of rho-associated coiled-coil-containing protein kinase 2 (ROCK2), has been approved by the FDA for the third-line treatment of cGvHD after ruxolitinib failure [12]. Belumosudil potentially targets cGvHD at two stages of pathogenesis: 1.) an anti-inflammatory rebalancing of the T helper 1 (Th1)/Th17 axis toward a regulatory T cell response by inhibiting signal transducer and activator of transcription 3 (STAT3) phosphorylation and upregulating STAT5 phosphorylation [12–14], and 2.) an anti-fibrotic effect by ameliorating profibrotic mediators such as tumor growth factor-β and preventing myofibroblast differentiation and collagen production [15, 16].

Two phase-II trials by Jagasia et. al. (KD025) and Cutler et al. (ROCKstar trial) presented overall response rates (ORR) of 65% and 74% after a median follow-up of 29 months and 14 months, respectively [17, 18]. The duration of response (DOR) was 35 and 54 weeks, respectively. These data are encouraging particularly given that both studies included extensively pretreated patients. The recently published 3-year follow up of the ROCKstar trial confirmed a high ORR of 74%, with 8% of the patients remaining on therapy for a minimum of three years, while 24% were completely off immunosuppression [19]. Two recent prospective trials from China and Japan provided similar results but were limited by the small number of patients included [20, 21]. The efficacy of belumosudil in the real-world setting is currently underresearched with the majority of data reflecting the experience of Northern American centers (Table 1). In light of this, a joint effort was made between five German and Swiss transplant centers (Regensburg, Hamburg, Kiel, Dresden, Basel) to analyze the efficacy and safety of belumosudil in the treatment of advanced cGvHD.

**Table 1:** Studies on the use of belumosudil for SR-/SD-cGVHD.

	*n*	design	treatment	prior rux %	LOTmed.	ORR %(time/best)	PR %	CR%	OS% (time)	DOR med., w	TTR	FSS%	Relapse% (n)	NRM%	FUmed.	organ response
Jagasia (2021) KD025 trial [17]	54	pro-mc phase 2a	bel	2	3	65 (best)	65	0	82 (2 y)	35w	>75% within 8w	76 (6 m) 47 (12 m)	13 (7)	-	29 m	best ORR*2 skin 30% eyes 50% mouth 70% liver 50% lungs 25% j/f 70% uGI 100% loGI 100% esop 75%
Cutler (2021) ROCKstar trial [18]	132	pro-mc phase 2	bel	29	3 (2–5)	72 (6 m)	70 (6 m)	2 (6 m)	89 (2 y)	54w	median 5w (4–66)	75 (6 m) 56 (12 m)	3	7	14 m	best ORR skin37% eyes 42% mouth 55% liver 39% lungs 26% j/f 72% uGI 53% loGI 69% esop 45%
Lee (2024) ROCKstar 3 y FU [19]	152	pro-mc	bel	-	-	74–76 (3 y)	-	-	-	-	-	44 (3 y)	-	-	-	-
Wang (2024) China [20]	30	pro-mc phase 2	bel	53	3	73	73	0	87 (1 y)	NR	median 4w	73 (6 m) 56 (12 m)	-	-	13 m	ORR skin 40% eyes 27% mouth -- lungs 15% j/f 78% uGI+ liver 67% loGI -- esop 60%
Inamoto (2024) Japan [21]	21	pro-mc phase 3	bel	0	1 (1–4)	86 (best)	86	0	95 (6/12 m)	NR (75% at 24w)	median 4w	NR	5 (1)	-	10 m	best ORR skin 55% eyes 20% mouth 67% lung 0%
Chin (2022) USA [30]	26	re sc	bel	50	3	77	-	-	100 (FU)	-	-	-	-	-	214 d	ORR skin 85% eyes 33% mouth 11% liver 13% lungs 30% j/f 53% loGI 0%
Raju (2024) USA [31]	66	re sc	bel	most	-	59 (6 m) 64 (12 m)	55 54	5 10	92 (6/12 m)	-	-	79 72	-	-	15 m	-
Raju (2024) USA [32]	14	re sc	bel-rux	93	-	71 (6 m) 70 (12 m)	57	14	-	-	-	-	-	0	14 m	ORR*3 skin 78% eyes 56% mouth 35% lungs 100% j/f 65% GI 80%
Caputo (2024) USA [33]	20	re sc	bel-rux	-	-	55	5	50	-	48 days	91 days	-	-	-	12 m	refer to [9]
Pusic 2024 USA [34]	14	re mc	bel-rux	100	3 (2–5)	43	43 (at FU)	0	-	-	median 3 m	-	0	7% (*n* = 1) at FU	11 m	ORR skin 21% eyes 14% mouth 21% lungs 7% j/f 14%

All values are rounded either in the original publication or for reasons of unification.

bel belumosudil, *CR* complete remission, *CS* corticosteroids, *DOR* median duration of response, esop esopsophagus, *FU* median follow-up, *FSS* failure-free survival, *GI* gastrointestinal involvement (upper/lower), *j/f* joints/fascia, *LOT* lines of therapy, median and range, *m* months, *mc* multicenter, *NR* not reached, *NRM* non-relapse mortality, *ORR* overall response rate, *OS* overall survival, *pat*. patient, *PR* partial remission, *pro* prospective, *prior-rux* patients with a history of ruxolitinib treatment, *pro* prospective, *ra* randomized, *re* retrospective, *rux* ruxolitinib, *sc* single-center, *w* weeks, *TTR* time to response, *y* years

“-“ no information published.

*early responses Fig. a3.

*2 estimated according Fig. a1.

**Fig. 1:**
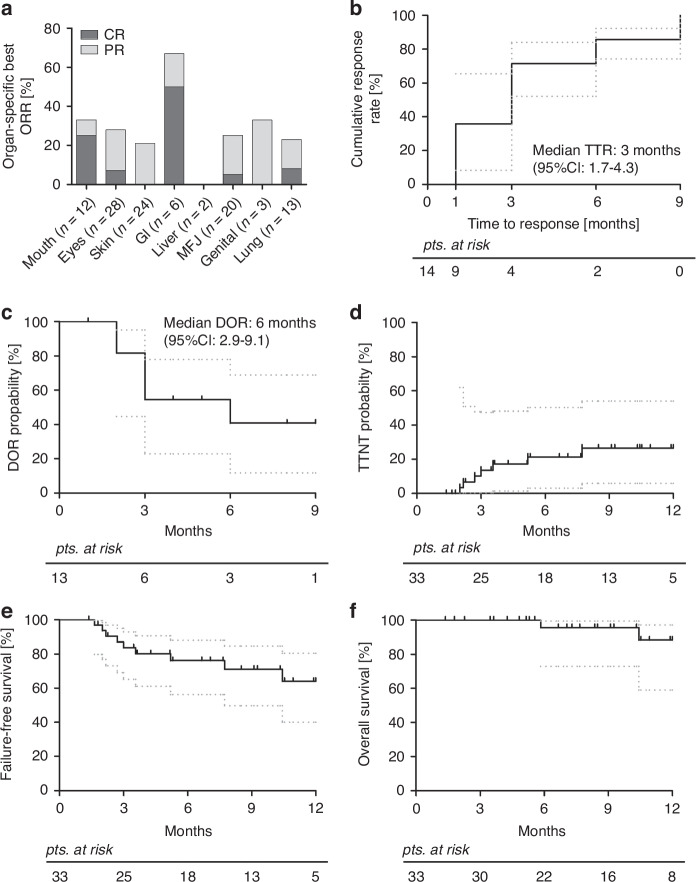
ORR by organ system and durability of response to belumosudil. **a** The individual organ-specific best objective response rate (ORR) comprising complete remissions (CR, dark gray) and partial remissions (PR, light gray); Kaplan-Meier curves of (**b**) time to response (TTR), **c** duration of response (DOR), **d** time to next treatment (TTNT), **e** failure-free survival (FFS), and (**f**) overall survival (OS). The gray dotted lines represent the 95% confidence interval (95%CI).

*3 estimated according Fig. 1

## Patients and Methods

### Patients

This multicenter, retrospective observational study was conducted at the allogeneic stem cell transplantation centers in Regensburg, Hamburg, Kiel, Dresden (Germany), and Basel (Switzerland). The study includes all consecutive adult patients (≥18 years) with persistent cGvHD who received belumosudil for at least 4 weeks (*n* = 33). One patient with atypical CNS-cGvHD was excluded from the final analysis due to the absence of reliable, clearly defined response criteria. The treatment with belumosudil had been requested on a patient-individual basis due to the lack of EMA approval. The study was conducted in accordance with the Declaration of Helsinki and was approved by the ethics committee at Regensburg (no. 23-3449-104). All patients alive gave informed consent.

### Treatment

Patients received 200 mg belumosudil daily *per os*, and dose was adjusted as recommended by the manufacturer (twice daily) if concomitant medication with proton pump inhibitors (PPI) was necessary. Concomitant immunosuppression (IS) was allowed but had to be initiated at least 1 month prior to belumosudil initiation and had to have failed to induce a response. Belumosudil was administered until progression of cGvHD, unacceptable toxicity, or expiration of insurance approval.

### Response assessment and efficacy endpoints

Organ-specific cGvHD assessment was performed according to the 2014 National Institute of Health (NIH) consensus criteria [22] at baseline and follow-up timepoints after 1, 3, 6, 9, and 12 months of treatment. Response assessment was discontinued if a new IS was initiated or the concurrent IS was increased in dose.

### Efficacy endpoints and safety

The response is reported as the best overall response rate (ORR) representing the rate of patients achieving complete (CR) or partial remission (PR) at any time point during the observational period. CR is defined as the resolution of all cGvHD manifestations, whereas PR indicates an improvement in at least one organ without progression in another organ [23]. Additionally, organ-specific responses (CR, PR, stable disease [SD], and progressive disease [PD]) are demonstrated. To acknowledge the fact, that treatment may result in a response not detectable by the NIH response criteria (i.e., reduction of body surface involved but remaining localized deep sclerosis), a subjective clinical response assessed either by the physician’s evaluation or patient-reported outcome is additionally reported. Secondary endpoints are time to response (TTR, defined as time between belumosudil onset and first CR or PR of cGvHD), duration of response (DOR, defined as the time from first CR or PR to subsequent progression of cGvHD), and corticosteroid (CS) dose reductions. The time-to-next-treatment (TTNT) covers the period from belumosudil initiation to the subsequent initiation of a new immunosuppression. Failure-free survival (FFS) is defined as the absence of new IS/significant dose increase of CS, relapse of the underlying disease, and/or death for any reason [24]. Overall survival (OS) is defined as the period from belumosudil initiation to the patient´s death. TTNT, FFS and OS are reported as percentages at 6- and 12-month follow-up. Treatment-emergent adverse events (TE-AE) of grade 3 or higher are evaluated and documented in accordance with the common terminology criteria version 5.0 (CTCAE).

### Statistical analysis

Statistical analyses were performed using SPSS Version 28.0.0.0 (IBM, Armonk, NY, USA) and PRISM 5 (GraphPad, San Diego, CA, USA). The level of significance was set at a two-sided *p* ≤ 0.05 with 95% confidence intervals. Baseline patient characteristics are demonstrated as frequency (*n* [%]) and median (range), ORR is reported as percentage of all patients. TTR, DOR, TTNT, FFS, and OS are depicted as Kaplan-Meier curves.

## Results

### Patient characteristics

A total of 33 consecutive patients who underwent treatment with belumosudil were identified and analyzed at five German and Swiss centers (Regensburg, Hamburg, Kiel, Basel, Dresden) between March 2022 and April 2024. As of the data cut-off date (2024/04/15) the median follow-up period was 8.5 months (range, 1.4–24.7 months).

The baseline characteristics are presented in Table 2. At initiation of belumosudil treatment, the median age was 59 years (range, 26–78 years). The treatment commenced at a median of 43.6 months (range, 8.9–256.3 months) following the onset of cGvHD. A median of four prior lines of systemic treatment (range, 1–11 LOTs) had been administered, including one second-line treatment in a patient who developed cGvHD (esophageal stenosis) during taper of prior aGvHD treatment with prednisolone, mycophenolate-mofetil, and tacrolimus. In total, 28 patients (85%) had received three or more prior lines of cGvHD treatment. Pretreatment included ruxolitinib in all but one patient and ibrutinib in nine patients (27%). Refractoriness to the prior treatment line was observed in 31 patients (94%) with 21, 11, and 1 patients showing steroid refractoriness, dependency, and intolerance, respectively. At the time of belumosudil initiation, 22 patients (67%) were receiving corticosteroid co-medication and 24 patients (73%) required concomitant immunosuppressive agents. At the treatment start, the NIH consensus cGvHD global severity score was moderate in two patients (6%) and severe in 31 patients (94%). The median number of organs involved was 3 (range, 1-6 organs), with the majority of manifestations involving the eyes (*n* = 27), skin (*n* = 24), and fascia (*n* = 20). Moreover, 12 patients had pulmonary cGvHD, with an organ score grade 3 in 8/12 patients. The dosage of belumosudil was 200 mg in all cases, except for two patients who required an adjustment due to PPI treatment. At last follow-up, treatment was ongoing in 20 patients (61%), the median duration of treatment was 7.1 months (range, 1.2–24.7 months), and 7 patients (21%) have received ≥ 12 months of belumosudil. The reasons for treatment discontinuation included cGvHD progression or lack of response (*n* = 8), adverse events (*n* = 2), sustained response (*n* = 2), and death (*n* = 1).

**Table 2:** Patient baseline characteristics.

Parameter	*n* (%)
Total	33 (100)
Male sex	20 (61)
Age at belumosudil start, years (range)	59 (26–78)
*Disease*	
AML/ALL	19(58)
Lymphoma	5 (15)
MDS	1 ((3)
MPN	8 (24)
*Donor constellation*	
MRD	13(39)
MUD	16(49)
MMUD	3 (9)
MMRD	1 (3)
Female donor/male recipient	8 (24)
* Graft source*	
PBSC	32(97)
BM	1 (3)
*Conditioning intensity*	
NMA	1 (3)
RIC	17 (52)
Standard/myeloablative	15 (46)
*GvHD prophylaxis*	
CNI	1 (3)
CNI/MMF	18 (55)
CNI/MTX	12 (36)
PostCy	1 (3)
ATG	15 (45)
Unknown	1 (3)
*Prior aGvHD*	25 (76)
Grade 0-1	14 (42)
Grade 2-4	19 (58)
*cGvHD onset*	
Classic	23 (70)
Overlap	10 (30)
De novo	8 (24)
Quiescent	15 (46)
Progressive	10 (30)
*Severity of cGvHD at onset/at belumosudil initiation*	
Mild	10 (30)/0
Moderate	11(33)/2 (6)
Severe	12 (36)/31(94)
*Organ involvement at onset/at belumosudil initiation*	
Mouth	21 (64)/12(36)
Eyes	11 (33)/27(82)
Skin	18 (55)/24(73)
Gastro-intestinal	4 (12)/6 (18)
Liver	9 (27)/2 (6)
Fascia/joints	2 (6)/20(61)
Genital	1 (3)/2 (6))
Lungs	3 (9)/12(36)
Steroid response	
Refractory	21(64)
Dependent	11(33)
Intolerant	1 (3)
*Concomitant IS at belumosudil initiation*	
Ruxolitinib	10(30)
MMF	3 (9)
Ciclosporin A	3 (9)
Tacrolimus	9 (27)
Extracorporeal photopheresis	6 (18)
Abatacept	2 (6)
Isotretinoin	1 (3)
Prior lines of treatment	4 (1–11)
Three or more prior LOTs	28(85)
Prior ruxolitinib	32(97)
Prior ibrutinib	9 (27)
Prior axatilimab	2 (6)
Treatment start after cGvHD onset, months (range)	43.7 (8.9–256.3)

*AML/ALL* acute myeloid/lymphoblastic leukemia, *MDS* myelodysplastic syndrome, *MPN* myeloproliferative neoplasm, *MRD/MUD* matched related/unrelated donor, *MMRD/MMUD* mismatched related/unrelated donor, *PBSC* peripheral blood stem cells, *BM* bone marrow, *NMA* non-myeloablative, *RIC* reduced intense conditioning, *CNI* calcineurin inhibitor, *MMF* mycophenolate mofetil, *MTX* methotrexate, postCy post-transplantation cyclophosphamide, *ATG* anti-thymocyte globulin, *aGvHD/cGvHD* acute/chronic graft-versus-host disease, *IS* immunosuppression, *LOT* lines of treatment.

### Response

The best overall response rate (ORR) was 42% (95%CI, 25-60%) with no significant differences observed in key subgroups (Supplementary Fig. 1). All overall responses were partial remissions (PR), yet complete organ remissions (CR) were achieved in patients with cGvHD manifestations of the mouth (*n* = 12, ORR = 33%, CR = 25%), eyes (*n* = 28, ORR = 29%, CR = 7%), GI tract (*n* = 6, ORR = 67%, CR = 50%), fascia (*n* = 20, ORR = 25%, CR = 5%), and lungs (*n* = 13, ORR = 23%, CR = 8%) (Fig. 1a). Of note, one patient each developed new ocular, pulmonary and genital cGvHD during treatment with belumosudil. The patient-specific organ responses (best response and response at last follow-up) are presented in the supplementary materials (Supplementary Fig. 2). Table 3 presents the response assessments conducted at the 1-, 3-, 6-, 9-, and 12-month follow-up time points. The median time to response (TTR) was three months (range, 1-9 months, 95%CI, 1.7–4.3 months) with 12 of 14 patients (86%) responding during the first six months (Fig. 1b). The median duration of response (DOR) was six months (range, 0-9 months, 95%CI, 2.9–9.1 months, Fig. 1c), which was ongoing in 8/14 patients (57%) at last follow-up. A subjective response, as determined by physician assessment or patient report, independent of reduction in NIH global severity or organ grading, was observed in 24 patients (73%). The modified Lee Symptom Scale [25] was assessed in only a fraction of patient and is therefore not reported. As compared to baseline at onset of belumosudil, steroid dose reduction was achieved in 14 of 22 patients with a median best reduction of 50% (range, 17-100%) including 8 patients with a reduction of at least 50% of initial dosage. Other concomitant immunosuppression was reduced or discontinued in 11 of 24 patients (Supplementary Table 1).

**Table 3:** Response assessment at indicated timepoints.

	1 month	3 months	6 months	9 months	12 months
Patients	*n* = 33	*n* = 30	*n* = 25	*n* = 22	*n* = 17
**PR** = **ORR**	**5 (15%)**	**8 (27%)**	**7 (28%)**	**6 (27%)**	**1 (6%)**
SD	25 (76%)	13 (43%)	8 (32%)	2 (9%)	3 (18%)
MR	1 (3%)	1 (3%)	2 (8%)	2 (9%)	2 (12%)
Not performed	1 (3%)	1 (3%)	0	3 (14%)	0
Excluded (new IS)	0	5 (17%)	8 (32%)	9 (41%)	10 (59%)

*PR* partial remission, *ORR* objective response rate, *SD* stable disease, *MR* mixed response, *IS* immunosuppression.

### TTNT, FFS, and OS

Given the limited median follow-up of 8.5 months, only the first 12 months after treatment initiation were considered for survival analysis. During this period, neither TTNT (Fig. 1d), nor FFS (Fig. 1e), and OS (Fig. 1f) reached the median. Nine patients (27%) required new IS resulting in a TTNT probability of 21% (95%CI, 3–50%) and 27% (95%CI, 6–54%) after 6 and 12 months, respectively. The FFS rates at 6 and 12 months were 76% (95%CI, 56–88%) and 64% (95%CI, 40–80%, Fig. 1e). The events that constituted failure were the initiation of a new IS in 9 patients and non-relapse mortality (NRM) of 1 patient. The OS rates at 6- and 12-months were 96% (95%CI, 73–99%) and 88% (95%CI, 59–97%) respectively, with three patients (9%) having died due to NRM during the observation period (Fig. 1f).

### Adverse events

A total of 11 TE-AE classified as CTCAE grade 3 or 4 occurred in nine patients (27%) with seven infectious and four non-infectious events (Table 4). In light of the retrospective nature of this real-world data analysis, it was not feasible to sufficiently capture CTCAE grade 1 and 2 AEs, and thus they are not presented here. Two patients required treatment discontinuation due to TE-AE: one patient experienced cardiac decompensation and influenza A pneumonia necessitating invasive ventilation, and the other patient developed retinal detachment. It is noteworthy, that both patients exhibited partial remission of cGvHD at the time of belumosudil discontinuation. One patient died while undergoing treatment with belumosudil, which was most likely due to pulmonary infection in a patient with severe obstructive lung involvement. Two additional patients died during the observation period at 80 and 282 days after cessation of belumosudil treatment (1 patient with progressive pulmonary cGvHD and 1 patient with pneumogenic sepsis). Moreover, no relapse of underlying malignancy was observed during treatment and follow-up.

**Table 4:** Treatment-emergent adverse events (CTCAE grade 3/4).

Patients	9 (27)
**Infectious**	7 (21)
- Pneumonia	6 (18)
- Soft tissue	1 ((3)
**Non-infectious**	4 (12)
*GI tract*	2 (6)
- Lower GI hemorrhage	1 (3)
- Esophagitis (LA grade D)	1 (3)
*Others*	2 (6)
- Retinal detachment*	1 (3)
- Heart failure/decompensation*	1 (3)

*GI* gastrointestinal, *LA* Los Angeles classification; *resulting in discontinuation of belumosudil treatment.

## Discussion

The treatment of steroid-refractory cGvHD remains a major challenge after allogeneic stem cell transplantation with a restricted number of approved agents available. To provide further insights into the efficacy and safety of the FDA-approved ROCK2-inhibitor belumosudil in a real-world patient cohort, we performed a retrospective analysis of 33 patients who had received belumosudil on an individualized salvage approach at five German and Swiss transplantation centers. The study population consisted of patients with predominantly severe cGvHD (*n* = 31) involving a median of 3 organs including difficult-to-treat advanced manifestations such as sclerotic skin (*n* = 24) and severe pulmonary cGvHD (*n* = 13). The initiation of treatment more than 40 months after the initial cGvHD diagnosis, a median of four prior treatment lines, and 94% of patients exhibiting refractoriness to the preceding therapy underscore the challenging study cohort.

Belumosudil yielded an ORR of 42% including complete organ remissions in all affected manifestations except for two patients with liver cGvHD. Of note, patients with gastrointestinal cGvHD manifestations including esophageal strictures and a history of long-lasting diarrhea preferentially responded to belumosudil treatment (ORR 67%, CR 50%), which is in line with previous reports [18]. Furthermore, de-escalation of concomitant immunosuppression (11/24 patients) and steroid dosage (8/22 ≥ 50% reduction) underline the efficacy of belumosudil. The median time to response of three months reflects the nature of predominantly fibrotic and sclerotic lesions in the involved patient population. A six-months duration of response with 20 patients (61%) remaining on treatment at the last follow-up is encouraging. Recognizing the facts, that 1.) response meeting the NIH criteria remission threshold [22] is often hampered by fixed and sclerotic manifestations [26], and 2.) that cGvHD significantly impairs quality of life [27], we also evaluated a subjective response based on informal patient reports and physician estimations. The subjective response rate of 73% distinctly outperformed the ORR, which highlights the challenges in evaluating clinically meaningful response in patients with advanced severe cGvHD.

Nevertheless, data from recent US [17, 18], Chinese [20], and Japanese [21] phase II trials indicated ORRs of 65 to 85% and a time to response of only 4 to 8 weeks, which was confirmed by a previously published 3-year follow-up of the US ROCKstar study [19]. The characteristics of our real-world cohort, which was selected for belumosudil salvage treatment after failure of a large number of best available alternative therapies, may at least partially explain the differences in ORR and TTR. Pulmonary cGvHD with an NIH lung score of 3 is often deemed unresponsive to treatment and irreversible [28] and was therefore excluded from other clinical trials, whereas we included eight patients with pre-existing severe pulmonary cGvHD. The high number of patients with moderate and severe pulmonary cGvHD also translated into a number of 94% of patients with severe cGvHD, whereas the aforementioned studies reported a percentage of 43 to 78%. Furthermore, compared to the other prospective trials the population presented here was the oldest (median 59 years versus 31–56 years) with the longest time from initial cGvHD diagnosis to belumosudil treatment (40 months versus 24-28 months). The cohort has received the highest number of prior LOTs (median 4, range 1-11) including ruxolitinib in all but one patient (97%). This differs from the prospective studies, which reported a median of 1–3 LOTs and a maximum of 50% of patients with prior ruxolitinib treatment [17, 18, 20, 21].

FFS was 76% and 64% at 6 and 12 months, respectively, which is highly consistent with previously reported cohorts. In 9 of 10 cases, the initiation of new immunosuppression was identified as the FFS event, which translated into a next treatment probability of 27% at 12 months. This is in line with a TTNT of 14 months reported by Jagasia and collaborators [17]. The OS rates after 6 and 12 months amounted to 96% and 88%, respectively. One death occurred due to multi-organ failure and severe sepsis on treatment with belumosudil, which might be considered treatment-related, whereas 2 further patients died due to non-relapse mortality after earlier discontinuation of the drug in the presence of refractory cGvHD.

Sclerotic and pulmonary manifestations represent highly morbid forms of cGvHD due to an inadequate response to treatment and irreversibility [26]. In our cohort, cGvHD of the skin with deep and/or superficial sclerosis was the most common manifestation (*n* = 24). Belumosudil induced an organ-specific NIH response of 21% (*n* = 5). The subjective response rate was notably higher, with 19 of the 24 patients exhibiting improvement in mobility and skin softening although below the NIH response threshold, including local partial or complete remission with grade 3 residual elsewhere. At belumosudil treatment onset, 12 patients showed pulmonary cGvHD with predominantly moderate (*n* = 3) und severe (*n* = 8) NIH lung scores. The lung-specific best ORR was 23% and a durable response was yielded in 2 of 3 patients with moderate lung involvement, whereas only a short-term partial remission was achieved in one patient with severe pulmonary cGvHD. This is in line with the results of a pooled analysis of the US belumosudil trials conducted by DeFilipp et al., which demonstrated a bronchiolitis obliterans syndrome-specific ORR of 32%. The response rate was inversely proportional to the severity of lung manifestations with no responses observed in patients with grade 3 pulmonary cGvHD [29].

Belumosudil exhibited a tolerable safety profile and TE-AEs ≥CTCAE grade 3 occurred in 27% of patients (*n* = 9). In contrast, ruxolitinib and ibrutinib induced up to 57% TE-AE in much-noticed phase II and phase III trials [5, 7]. The observed preponderance of respiratory infections (*n* = 6) in patients treated with belumosudil has also been reported by others [17, 18] and should be considered in patients with pre-existing lung involvement. Although frequently described, only two patients in our cohort suffered from gastrointestinal adverse events, of whom one patient developed severe esophagitis as a consequence of discontinued PPI treatment. No relevant hematological adverse events have been observed. Only two patients discontinued treatment due to adverse events (retinal detachment, heart failure).

We acknowledge the limitations of our study due to its retrospective nature, the short follow-up, and the small number of patients included. Future phase-II and -III trials are expected to provide data on the safety and efficacy of belumosudil in specific patient populations with cGVHD (NCT05567406), the use in first-line therapy (NCT05996627) or new onset and incipient bronchiolitis obliterans syndrome (NCT05922761), in combination with steroids (NCT06143891, ROCKnrol-1) or rituximab (NCT06046248).

In conclusion, the results demonstrated a promising ORR and FFS with an acceptable safety profile in the German and Swiss real-world cohort. The noteworthy efficacy and steroid-sparing effect of belumosudil observed in a heavily pretreated patient cohort with an advanced cGvHD profile including highly morbid forms provide the rationale to evaluate belumosudil in earlier treatment lines.

## Supplementary information

Supplementary material

## Data Availability

The data will be made available at any time upon personal request.
