# Supplementary material for: Safety and efficacy of the ROCK-2-inhibitor Belumosudil in cGvHD treatment - a retrospective, German-Swiss multicenter real-world data analysis
Source: Bone Marrow Transplant. 2025 Jan 14;60(4):439–46. doi: 10.1038/s41409-024-02507-9 (PMC11971036; doi:10.1038/s41409-024-02507-9)
Supplement: Supplementary file 1 — Supplementary material [file 41409_2024_2507_MOESM1_ESM.docx]

**Supplementary Table 1.** Concomitant immunosuppression/steroids at onset of belumosudil treatment. Numbers in brackets indicate the timepoint of discontinuation of the specific agent.

| Patient | Concomitant immunosuppression | Concomitant  steroids | Reduction of  steroids by x% |
| --- | --- | --- | --- |
| 1 | Ruxolitinib (6m) | - |  |
| 2 | MMF | - |  |
| 3 | CsA, Ruxolitinib (3m), ECP | - |  |
| 4 | Ruxolitinib, ECP (3m) | - |  |
| 5 | CsA | yes | 38 |
| 6 | Ruxolitinib | yes |  |
| 7 | CsA | yes |  |
| 8 | Ruxolitinib (1m) | yes |  |
| 9 | Ruxolitinib (1m) | - |  |
| 10 | - | - |  |
| 11 | Ruxolitinib (1m) | - |  |
| 12 | Ruxolitinib (1m) | - |  |
| 13 | Tacrolimus, MMF (12m), ECP | yes |  |
| 14 | Tacrolimus, ECP | - |  |
| 15 | Tacrolimus | yes | 100 |
| 16 | ECP | yes |  |
| 17 | Tacrolimus, ECP | yes | 40 |
| 18 | MMF | yes |  |
| 19 | Ruxolitinib | - |  |
| 20 | Ruxolitinib, Abatacept (1m) | yes | 67 |
| 21 | Tacrolimus | yes | 25 |
| 22 | - | yes | 50 |
| 23 | - | yes | 50 |
| 24 | Tacrolimus, Abatacept (3m) | yes | 50 |
| 25 | - | - |  |
| 26 | - | yes | 83 |
| 27 | Tacrolimus | yes |  |
| 28 | - | yes | 33 |
| 29 | - | yes | 17 |
| 30 | - | yes | 50 |
| 31 | Tacrolimus, Isotretinoin | yes | 67 |
| 32 | - | yes | 40 |
| 33 | Tacrolimus (6m) | yes |  |

**Supplementary Figure 1.** Forrest-plot of objective response rates (ORR) in key subgroups. No significant differences were found. *Abbreviations*: no. = number

**B**

**A**

*Abbreviations*: NIH = National Institute of Health, GI = gastrointestinal, MFJ = muscle/fascia/joints, CR = complete remission, PR = partial remission, SD = stable disease, MR = mixed response, PD = progressive disease, new = new onset during treatment, 95%CI = 95% confidence interval.

**Supplementary Figure 2.** Patient-individual organ-specific response (ORR). (A) Best response and (B) response at last follow-up. The numbers in brackets indicate the organ-specific cGvHD grade at the initiation of belumosudil treatment.
